# Research lessons during the COVID-19 pandemic: collecting longitudinal physical and mental health outcomes

**DOI:** 10.1186/s13690-021-00781-3

**Published:** 2022-01-04

**Authors:** Kristen Grove, Megan Harrold, Sheeraz Mohd, Varsha Natarajan, Elizabeth Hurn, Jane Pearce, Vinicius Cavalheri, Carol Watson, Dale W. Edgar, LATER-19 Coinvestigator Group, Andrew Maiorana, Angela Jacques, Ann White, Caitlin Vicary, Carol Watson, Caroline Roffman, Emma-Leigh Synnott, Hui Jun Chih, Ian Suttie, Ivan Lin, Jade Larsson, Jessica Tearne, Linda Woodhouse, Lisa van der Lee, Louise Naylor, Mercedes Elliott, Paul Gittings, Peta Winship, Robyn Timms, Sheldon Wulff, Tracy Hebden-Todd

**Affiliations:** 1 Department of Physiotherapy, Royal Perth Hospital, Royal Perth Bentley Group, East Metropolitan Health Service, Perth, Western Australia; 2 Curtin School of Allied Health, Curtin University, Bentley, Western Australia; 3 Department of Cardiology, Fiona Stanley Hospital, Murdoch, Western Australia; 4 Department of Physiotherapy, Fiona Stanley Hospital, South Metropolitan Health Service, Murdoch, Western Australia; 5 Department of Physiotherapy, Sir Charles Gairdner Hospital, North Metropolitan Health Service, Nedlands, Western Australia; 6 Consumer Advisory Council, Fiona Stanley Fremantle Hospital Group, South Metropolitan Health Service, Murdoch, Western Australia; 7 Western Australian Health Translation Network, Perth, Western Australia; 8 Burn Injury Research Node, The University of Notre Dame Australia, Fremantle, Western Australia; 9 Division of Surgery, Medical School, University of Western Australia, Crawley, Western Australia; 10 Fiona Wood Foundation, Fiona Stanley Hospital, Murdoch, Western Australia

**Keywords:** Research participation, Public engagement, COVID-19, Longitudinal, Physical recovery, Mental health

## Abstract

**Background:**

Participant enrolment, assessment and/or delivery of intervention in many clinical trials during the COVID-19 pandemic were severely impacted by public health measures limiting physical contact. This report describes the lessons learned in completing a repeated measures cohort study involving suspected and confirmed COVID-19 survivors at three sites in Perth, Western Australia.

**Main body:**

An observational analysis of the conduct and data completeness results of the LATER-19 trial. People with COVID19 symptoms who were tested between February and November 2020 were recruited. In both those who tested positive and those who tested negative (control group) for COVID19, data on physical function and mental health were collected at two time points up to eight months after COVID19 testing. Recruitment of the controls was targeted from hospital records for comparison, it was balanced for age and sex and for the non-hospitalised group also comorbidities.

A sample of 344 participants was recruited: 155 (45.1%) COVID-19 positive. Taking the research design and environmental adaptations into account, we recorded > 90% participant engagement during the trial. Of the 637 planned assessments, objective measures were completed on 602 (94.5%) occasions; 543 (90.2%) were on-site and 59 (9.8%) were remote. A total of 577 (90.6%) mental health/symptoms surveys, 569 (89.3%) 1-min sit-to-stand tests, and 520 (81.6%) handgrip strength tests were completed.

**Conclusion:**

The sample size and high completion rate of planned assessments during the LATER-19 trial potentially increases the contextual, groupwise generalisability of the results. The results demonstrate the effectiveness of a simple, rapid, reproducible and adaptable battery of assessments, leveraging telehealth and digital solutions.

**Trial registration number:**

Australian and New Zealand Clinical Trial Registration (ANZCTR): ACTRN12621001067864.

## Background

During the COVID-19 pandemic, the rapidly burgeoning biomedical research sector was in stark contrast to the negative impact of COVID-19 on clinical trials [1]. By April 2021over 1700 clinical trials were suspended due to barriers around requirements for in-person contact in enrolment, assessment and/or intervention [2], while many planned drug trials examining COVID-19 treatments delivered results with minimal usefulness [3]. With the spotlight on research integrity [4], caution needs to be applied for conclusions drawn from results lacking longitudinal data or a control group [5, 6]. Trials that have successfully persevered over the last year, have implemented research designs that are streamlined, accessible, adaptable, and utilised remote engagement appropriate to the environment and subject [2, 3]. In this report we present the Life AfTER COVID-19 (LATER-19) researchers’ experience in Western Australia (WA), which supported the engagement in a longitudinal observational cohort study designed specifically for completion during the pandemic. Currently, the results of the 12-month, third repetition of assessments are being finalised.

### Western Australian context

Western Australia is a state very familiar with the tyranny of distance and its negative impact on health outcomes. With the advent of COVID19, a time when a high density of population and close personal proximity is a burden to public health measures, this tyranny of distance came wrapped as a blessing. With a land area greater than 2.5million km^2^ and a population density of only 0.89 persons/km^2^, finding innovative and reliable solutions for timely health assessments and follow up, regardless of location or destination of discharge, has been the norm for clinicians. Despite being able to leverage available communication infrastructure, the challenge in resourcing and establishing research in the context of an infectious disease pandemic remained. Initial application for grant funding was submitted in April 2020. This time point was characterised by climbing daily case rates and peaking hospital admissions locally and internationally. With the impact of the COVID-19 pandemic emergent in jurisdictions outside Australia, the relative isolation and low population density of Perth was uniquely beneficial in allowing substantial preparation time. Further, the WA health care sector was fortunate not to be overburdened due to a highly successful border management and quarantine processes; rapid-uptake and adherence to social-distancing rules by community at large; and, subsequent very low community spread. The fortunate low rate of disease transmission afforded the LATER-19 team an opportunity to design, test and set up research with a view to inform solutions to assessing and treating physical and mental health recovery in the challenging pandemic environment.

The aim of this paper is to reflect on and share the lessons learned with future and current clinician and public health researchers. This report presents the key factors that contributed to the capacity to conduct, and longitudinal data completeness, in the LATER-19 trial.

## Method

The LATER-19 Trial was conducted primarily at three tertiary hospitals in Perth, WA. The original study design has been previously described [7]. The original design was aimed at analysing the trajectory of the participant journey during and post inpatient stay with the goal of predicting clinical deterioration and/or requirement for inpatient rehabilitation. To achieve this, the hospitalised participant would at 48 h intervals complete a 1-min sit-to-stand test (1STS) [8], handgrip strength via JAMAR dynamometer, and the Edmonton Symptom Assessment Scale (ESAS). The 1STS required the participant to complete their maximum number of sit to stand repetitions in sixty seconds. In addition to the usual protocol [8] LATER-19 measured heart rate (HR) and peripheral oxygen saturation (SpO_2_) and participant-rated breathlessness and leg fatigue was measured via the modified Borg Rating of Perceived Exertion scale. The ESAS provided a snapshot of breathlessness and fatigue symptoms on a 10 point scale.

Follow-up was planned via repeated measure at 3 months, 6 months and 12 months. Physical function was assessed via: 1STS and handgrip strength. Validated surveys [7]: Impact of Events Scale-6 (IES-6), Hospital Anxiety and Depression Scale (HADS), Fatigue Severity Scale (FSS), Euro-Qol: EQ-5D-5L and Modified Medical Research Council Dyspnoea Scale (mMRC) assessed Post Traumatic Stress Disorder, anxiety and depression, quality of life, fatigue and breathlessness respectively.

**Fig. 1:**
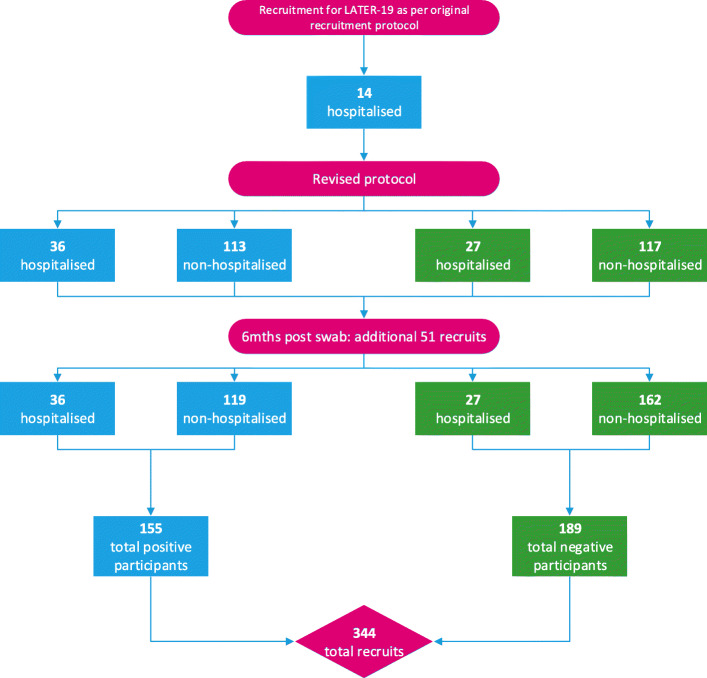
LATER-19 Recruitment

Recruitment included adults ≥18 years, initially targeting only those admitted to one of the 3 hospital site with a positive result on a reverse transcription polymerase chain reaction (PCR) serology test. However the unexpected boon of low case numbers requiring hospital admission necessitated a pivot in scope (see Fig. 1). WA State directions at the time targeted PCR testing to those displaying respiratory symptoms and/or a fever, thus a negative result by PCR in this situation satisfied the term ‘suspected COVID19’, providing an opportunity for recruitment of a control group. Ethics and Governance amendments expanded recruitment to include suspected COVID19 cases regardless of admission. This enabled a more detailed analysis of the broader COVID19 situation in WA, improving the generalisability of results [9] particularly for comparison to other low severity COVID19 cohorts. All those advised of a positive result were eligible to be contacted. Recruitment of the control group was targeted. Those non-hospitalised with a negative result were screened to match by sex, age and respiratory comorbidities. Those hospitalised with a negative result were screened to match by age (+/− 2 years) and sex only, as it was not feasible to match for comorbidities due to the very small cohort size. Participants were excluded if they had a pre-existing mental health illness, significant communication or cognitive impairment thought to impact their ability to complete the self-reported measures, or pre-existing condition preventing completion of the physical measures such a neuromuscular disorder or bony injury.

Site specific Standard Operating Procedures (SOPs) were developed for infection control, gaining consent, and completing physical assessments. Bespoke guidelines for commencing or ceasing 1STS were developed, tailored for safe remote assessment without in-room physical support. Pilot trials at two hospitals were completed to standardise the methods and inter-rater reliability between all three recruitment sites.

## Results

Recruitment before protocol amendment was 14 positive inpatients (see Fig. 1). Subsequently minimal inpatient data were collected. At first assessment timepoint (median 5.65 months), recruitment was 293. Due to timing of the amendment in relation to waning COVID19 cases a further 51 people were recruited after that time point, netting a total sample of 344 participants by second assessment timepoint (median 7.56 months). See Fig. 1 for recruitment flow. Of the 344 participants: 63 (18.3%) required admission to hospital, 155 (45.1%) of participants were COVID-19 positive, 201 (58.4%) were female, 328 (95.3%) resided in the Perth metropolitan region, 2 (0.6%) resided >150kms from Perth, 12 (3.5%) resided > 2500 km from Perth, 2 (0.6%) were internationals. See Table 1 for distribution of engagement in the total 637 possible post-acute assessments to eight months. Of the 602 assessments completed, 543 (90.2%) were completed on-site, and 59 (9.8%) were delivered remotely (via phone or telehealth), see Table 2.

**Table 1:** Participant engagement, combined 1st and 2nd data collection time points, *n* = 637 possible occasions

	Completed	%
**Assessment attendances**	602	94.5%
**Surveys completed**	577	90.6%
**1STS test**	569	89.3%
**1STS HR (max)**	532	83.5%
**1STS SpO**_**2**_	514	80.7%
**Handgrip strength**	520	81.6%

**Table 2:** Comparison of number of assessments completed on-site (543) and remotely (59), *n* = 602

	On-site	%	Remote	%	TOTAL	%
**Surveys complete**	531	97.8%	46	78.0%	577	95.8%
**1STS test**	516	95.0%	53	89.8%	569	94.5%
**1STS HR (max)**	512	94.3%	20	33.9%	532	88.4%
**1STS SpO**_**2**_	510	93.9%	4	6.8%	514	85.4%
**Handgrip strength**	520	95.8%	0	0.0%	520	86.4%

### Streamlined and accessible by design

The methods were developed through a process of rapid consultation, amassing experts in respiratory care, with consumer input sought during this planning. The assessment battery was derived through a robust process to establish consensus between experienced academics and clinicians, undertaken at a time when infection control policy was changing almost daily. Early identification of the need to pragmatically capture short and long-term data pertinent to holistic, physical and mental health recovery was key to the configuration of the standardised battery valid for a disease propagated within the respiratory system [10].

To minimise risk of transmission, the LATER-19 researchers designed innovative methods [7] to promote completion of physical components with minimal physical contact or specialised equipment. Assessments in the inpatient setting utilised a standard oximeter and in-room furniture. Patients could be recruited, and physical measures observed though the room’s window supplemented by phone contact, or bundled as part of usual care, assisted by clinicians entering the environment in personal protective equipment (PPE). For the sustainability of clinical resources, study methods were planned to minimise the use of PPE for research purposes. Assessment of grip strength via dynamometer was achieved by allocating them as single patient use per room, and possible only due to research funding enabling purchase of multiple extra devices (*n* = 5 per site). The simplicity of recruitment and study methods ensured low participant and clinician burden in the hospital environment, benefiting engagement. The array of survey measures were finalised to maximise follow-up regardless of patient’s capacity to physically assess or interact with them, using either paper-based or digital format dependent on patient circumstances.

### Adaptable process

As the original research design did not rely on direct physical contact, the standardised assessment could be reliably translated across a range of settings with minimal data loss. Assessments were successfully completed in the inpatient environment, outpatient department, and via phone/telehealth, the choice of which was tailored to patient circumstances. WA’s well-established telehealth system, developed to overcome the state’s extreme distance and remoteness, was leveraged as key study infrastructure to enhance protocol adherence, quality of data capture, and reduce missing data.

### Remote solutions

Digital platforms and telehealth solutions were invaluable in facilitating engagement, and managing risk of disease transmission. Use of the REDCap data management system allowed e-consent and e-survey response, one site recording the uptake of e-consent at > 70, and 98% utilising e-survey. We acknowledge the work of the Perth node of the International Severe Acute Respiratory and Emerging Infection Consortium trial (ISARIC) for pioneering the local process and establishing e-consent modules.

A flexible mode of review ensured physical location, travel restriction or work commitments were minimised as a barrier to engagement. On 20 occasions, participants were able to successfully complete the 1STS with HR measure remotely utilising their smartwatch. One while located in the Middle East and others at home or at their workplace. Two participants were able to complete 1STS with HR and SpO_2_ measure via phone call by accessing a local pulse oximeter, one in-situ at their metropolitan GP clinic, and another in their home interstate.

Telehealth or phone appointments accounted for 9.8% of assessments, resulting in missing items of HR, SpO_2_ and grip strength (with exceptions as above) (Table 2). This was balanced by capturing survey data and 1STS test in participants who would have otherwise gone un-recruited. Benefits for video links included a more personal touch, analysis of facial expression, body language, physical impairment and risk. However, these benefits were occasionally thwarted by issues of connectivity and continuity. In most cases issues were rectified by continuing assessment via phone call. Choice of phone or video telehealth was adjusted for the participant’s personal situation [2], accounting for access and familiarity with technology, especially in elderly or disadvantaged populations.

### Factors aiding longitudinal protocol adherence

The study follow-up rate was boosted by an eagerness to participate regardless of the participant’s PCR result, reflecting a strong intrinsic community motivation to assist in a situation of global uncertainty. During recruitment, a control participant was so eager to assist that she consented immediately and volunteered to attend the hospital in-person only a few hours after first phone contact was made. Despite being offered the option of a remote appointment, on many occasions, participants working fulltime external to hospital sites chose to take time from their lunchbreaks to attend the hospital in-person to facilitate complete assessments. Also, the two participants based in regional WA preferred to drive > 150 km to Perth, to be able to attend their appointments in-person.

### When research meets everyday life

In addition to the standardised instructions, when completing the 1STS remotely the participants were asked to say ‘sit’ every time they came in contact with the chair regardless of whether they were using video linkup or solely phone contact. This enabled the researcher to count repetitions irrespective of any issues with continuity of visual feed. This method also accommodated a participant suffering memory deficit post COVID-19. He had forgotten his scheduled assessment via phone call, and was out shopping. The LATER-19 protocol, participant and researcher were sufficiently flexible to avoid rescheduling as he voluntarily completed the 1STS assessment successfully on a nearby bench seat.

## Conclusion

Due to a unique situation the LATER-19 team has designed and successfully implemented a methodology for longitudinal follow-up of mental health and physical outcomes to suit a pandemic environment. Robust selection of valid, multimodal assessment tools in a collaborative approach was integral to the overall adaptable design, while eagerness to participate underpinned the high retention rate. The methodology framework presented can be rapidly replicated and translated into different situations in keeping with various infection control environments including inpatient, outpatient and telehealth settings.

## Data Availability

The datasets generated and/or analysed during the current study are not publicly available as publication of clinical datasets was not included in application for ethics approval. De-identified datasets are available from the corresponding author on request, which involves the execution of a data-sharing agreement.

## Abbreviations

1STS: 1 min sit to stand test
FSS: Fatigue severity scale
HADS: Hospital anxiety and depression scale
HR: Heart rate
IES-6: Impact of events scale-6
ISARIC: International severe acute respiratory and emerging infection consortium
LATER-19: **L**ife **A**f**TER** COVID**19** trial
mMRC: Modified medical research council dyspnoea scale
SOP: Standard operating procedures
SpO_2_: Peripheral oxygen saturation
WA: Western Australian
WAHTN: Western Australian health translation network
